# Trends and Complication Rates in Ulcerative Colitis Patients With and Without Helicobacter pylori Infections

**DOI:** 10.7759/cureus.37345

**Published:** 2023-04-09

**Authors:** Aaron Kahlam, Ayham Khrais, Ali Khalessi, Sushil Ahlawat

**Affiliations:** 1 Internal Medicine, New Jersey Medical School, Newark, USA; 2 Gastroenterology and Hepatology, New Jersey Medical School, Newark, USA; 3 Gastroenterology and Hepatology, Downstate Health Sciences University, Brooklyn, USA

**Keywords:** inflammatory bowel disease, immunology, microbiome, ulcerative colitis, helicobacter pylori

## Abstract

Background

Previous studies have shown an inverse relationship between ulcerative colitis (UC) and Helicobacter pylori infections (HPI). Though these two conditions have opposite geographic distributions, there may also be a physiological explanation for the decreased incidence of H. pylori infections in patients with UC. The purpose of this study is to analyze trends and complication rates of ulcerative colitis patients with and without HPI.

Materials and methods

The National Inpatient Sample (NIS) database was queried for patients with a primary diagnosis of UC, stratified by the presence of H. pylori infection. Patient demographics, length of stay, total hospital charges, and mortality were compared by H. pylori status. Additionally, complication rates were also compared between the two groups. Chi-squared and independent t-tests were used to compare outcomes and demographics, and multiple logistic regression was used to analyze primary and secondary outcomes.

Results

Patients with UC and HPI had a lower mortality rate (8.22 vs. 3.48, P<0.05, adjusted odds ratio [AOR] 0.33) and lower hospital charges ($65,652 vs. $47,557, p<0.05, AOR 1) with similar length of stay. Patients with UC and HPI also had lower rates of intestinal perforation (2.16% vs. 1.12%, p=0.05, AOR 0.408) and intrabdominal abscess formation (0.89% vs. 0.12%, AOR 0.165, p=0.072), though this difference was not significant. From 2001 to 2013, the incidence of UC has increased while the incidence of HPI has decreased.

Conclusions

The lower hospital charges and mortality rate as well as decreased rates of intestinal perforation and abscess formation suggest that there may be a physiologic role that HPI plays in modulating UC. Further studies into the interaction of these two conditions would be beneficial in clarifying their relationship and may help guide treatment of UC.

## Introduction

Ulcerative colitis (UC) is a relapsing-remitting form of inflammatory bowel disease (IBD) confined to the colon. Symptoms of UC may be limited to gastrointestinal manifestations and include abdominal pain, hematochezia, and diarrhea with urgency and tenesmus [1,2]. More severe systemic manifestations may include weight loss, anemia, venous thromboembolism, fever, and fulminant colitis with megacolon and perforation. Additionally, some patients may develop extra-intestinal manifestations which include IBD-associated arthropathy and ankylosing spondylitis, uveitis, erythema nodosum, pyoderma gangrenosum, and primary sclerosing cholangitis [1-3]. Contrarily, Crohn’s disease may present in any part of the gastrointestinal tract and includes transmural inflammation with fistula and stricture formation.

Ulcerative colitis has an incidence of 9-20 cases per 100,000 individuals and is slightly more prevalent than Crohn’s disease, with both being more prevalent in industrialized regions including the United States and Europe [1,2]. This uneven geographic distribution of IBD among regions at different stages of industrial development is hypothesized to be secondary to changing environmental exposures and dietary habits [2,3]. For example, patients from industrialized regions often consume a diet higher in fat and refined sugar, leading to changes in their gut microbiome that have been linked with the development of UC [2,4]. In their study, Vangay et al. demonstrated an immediate loss of microbiome diversity and function among Thai immigrants to the United States as they adapted to a Western diet. This decrease in diversity and function then became more pronounced among subsequent generations as the microbiome of those studied began to resemble more closely those of their European and American counterparts [5]. Other environmental factors affecting the microbiome have also been linked to the development of UC, including infection with pathological organisms such as Salmonella and, paradoxically, reduced exposure to other pathogens. The latter is thought to be due to imposed limitations on the development of the immune system, leading to abnormal response upon initial exposures to pathogenic microorganisms later on [2].

Helicobacter pylori (HP) is an example of a pathological organism. It is a gram-negative, urease-producing organism that colonizes the mucosa of the upper GI tract. Unlike UC, low socioeconomic status is associated with greater risk for HP infection, likely due to poor hygiene and consumption of colonized food products [6]. A multitude of pathogenic factors enable infection, including urease which produces ammonia that neutralizes the acidic gastric environment, and flagella which enable motility and colonization [7]. Through these mechanisms HP can colonize the gastric and duodenal mucosa, resulting in inflammation and mucosal erosion and ulceration. However, due to improved hygienic practices including cleaner drinking water and increased antibiotic use, HP prevalence has been decreasing globally [7,8].

Previous studies have established an inverse correlation between rates of HP infections (HPI) and UC in particular [9-11]. Over the past several years the incidence of UC has been increasing while the incidence of HP has been decreasing. This difference could be attributed to increasing industrialization, resulting in dietary modifications that predispose patients to UC as well as increased sanitation which is helping prevent HPI. However, as mouse models have demonstrated, an immunomodulatory effect of HPI may also be playing a role in inhibiting the development of UC. In this study, we aim to further explore this relationship by examining rates of UC complications in patients with and without HP. We hypothesize that if a mechanistic link is behind the observed differences, both the incidence and severity of UC would be impacted by HPI, while a purely epidemiologic cause would only impact incidence. To measure severity, we used the primary outcomes of hospital cost, length of stay and mortality, with secondary outcomes including complications related to UC. 

## Materials and methods

Data source

Data for this study was obtained from the National Inpatient Sample (NIS), the largest public all-payer inpatient database containing information on more than 7 million hospital stays in the United States. Developed by the Agency for Healthcare Research and Quality, the NIS contains no patient or hospital identifiers, and provides a nationally representative database of information. Data in the NIS represents 20% of all discharges from community hospitals within the United States. Sampling weight is applied annually, enabling precise national estimates. The NIS was investigated for hospitalized patients from 2001 to 2013 using the International Classification of Diseases, Ninth Revision, Clinical Modification (ICD-9 CM) codes to identify admitted patients with ulcerative colitis (UC) who also had a diagnosis of Helicobacter pylori infection.

Study design

This cross-sectional study utilized ICD-9 CM codes to identify admitted patients >18 years old with ulcerative colitis. These patients were then stratified based on the presence of the ICD-9 CM code for Helicobacter pylori to create two groups: patients with UC who did not have Helicobacter pylori (non-HP group) and those with UC who had Helicobacter pylori (HP group). Primary outcomes included inpatient mortality, length of stay (LOS), and hospital charges. Secondary outcomes included intestinal manifestations of UC (lower gastrointestinal bleed [LGIB], bowel obstruction, intra-abdominal abscess, intestinal perforation), extra-intestinal manifestations of UC (IBD-associated arthropathy, erythema nodosum, uveitis, primary sclerosing cholangitis [PSC]), and potential required surgical interventions for severe disease (ileostomy, colostomy). Primary insurance payer and median household income were also analyzed for each group. To assess the yearly prevalence of both UC and HP from 2001 to 2013, the NIS database was queried separately for patients with UC and patients with HP.

Statistical analysis

IBM SPSS Statistics 24 (IBM Corp., Armonk, NY, USA) was used to conduct statistical analyses. Outcomes and demographic data for both tested groups were assessed with independent t-tests and Chi-squared tests. Multiple logistic regression was utilized to characterize primary and secondary outcomes among both groups while controlling for age, gender, and race. Statistical significance was indicated with a p-value <0.05. Adjusted odds ratios (AOR) and associated 95% confidence intervals (CI) were calculated. Microsoft Excel (Microsoft Corp., Redmond, WA, USA) was utilized to create a scatter plot of the yearly prevalence of UC and HP, with a line of best fit generated for each variable, followed by calculation of relevant equations and relative risk (RR) values.

## Results

Among patients hospitalized from 2001 to 2013, 406,296 had a diagnosis of UC, 805 of whom were also diagnosed with Helicobacter pylori (Table 1). Mean age was similar among both non-HP and HP groups (58.35 years [SD=19.25] versus 59.77 years [SD=18.984]; p=0.564), with statistical significance only after adjustment for confounding variables (adjusted p<0.05; AOR 0.992; ACI 0.988-0.996), as shown in Table 1. Members of both groups were mostly female (non-HP: 55.14%, HP: 58.01%; p=0.101, adjusted p=0.166; OR 0.403 [CI 0.276-0.588], AOR 0.33 [ACI 0.212-0.512]), however comparison of sex at birth between both groups carried no statistical significance. In terms of racial distribution, both groups were predominantly Caucasian, with the non-HP group containing more Caucasians when compared to the HP group (77.2% versus 55.71%), shown in Table 1. The second most common race in both groups was Black (non-HP: 10.14% versus HP: 19.6%), followed by Hispanic (non-HP: 7.81% versus HP: 16.98%).

**Table 1:** Demographic data for ulcerative colitis patients with and without Helicobacter pylori infection n: sample size, SD: standard deviation

		Ulcerative colitis without Helicobacter pylori (n=405,491)	Ulcerative colitis with Helicobacter pylori (n=805)
Mean Age in Years (SD)		58.35 (19.25)	59.77 (18.98)
Sex	Male (%)	181,797 (44.86)	338 (41.99)
	Female (%)	223,442 (55.14)	467 (58.01)
Race	Caucasian (%)	256,597 (77.2)	361 (55.71)
	Black (%)	33,699 (10.14)	127 (19.6)
	Hispanic (%)	25,971 (7.81)	110 (16.98)
	Asian/Pacific Islander (%)	6,157 (1.85)	29 (4.47)
	Native American (%)	1,561 (0.47)	4 (0.62)
	Other (%)	8,388 (2.52)	17 (2.62)

Rates of UC complications are included in Table 2. Mortality was higher among patients with UC without a diagnosis of HP compared to those with HP (8.22% versus 3.48%; p<0.05, adjusted p<0.05; OR 0.403 [CI 0.276-0.588], AOR 0.33 [ACI 0.212-0.512]).

**Table 2:** Comparison of mortality, length of stay, hospital cost and IBD complications between ulcerative colitis patients with and without Helicobacter pylori infection IBD: inflammatory bowel disease; n: sample size; OR: odds ratio; CI: 95% confidence interval; AOR: adjusted odds ratio; ACI: adjusted confidence interval; n/a: not applicable; SD: standard deviation.

	Ulcerative colitis without Helicobacter pylori (n=405,491)	Ulcerative colitis with Helicobacter pylori (n=805)	Odds Ratio (CI)	p-value	AOR (CI)	Adjusted p-value
Mortality (%)	33,296 (8.22)	28 (3.48)	0.403 (0.276 to 0.588)	<0.05	0.33 (0.212 to 0.512)	<0.05
IBD-associated arthropathy	709 (0.17)	0 (0)	0.998 (0.998 to 0.998)	0.235	n/a	n/a
Erythema nodosum	330 (0.08)	0 (0)	0.998 (0.998 to 0.998)	0.418	n/a	n/a
Uveitis	182 (0.04)	1 (0.12)	2.77 (0.388 to 19.794)	0.289	2.964 (0.413 to 21.283)	0.28
Primary sclerosing cholangitis	5,201 (1.28)	3 (0.37)	0.288 (0.093 to 0.895)	<0.05	0.394 (0.126 to 1.227)	0.108
Lower gastrointestinal bleed	21,942 (5.4)	82 (10.19)	1.983 (1.577 to 2.492)	<0.05	1.972 (1.525 to 2.551)	<0.05
Bowel obstruction	8,785 (2.17)	16 (1.99)	0.916 (0.558 to 1.503)	0.728	1.073 (0.643 to 1.793)	0.786
Intra-abdominal abscess	3,608 (0.89)	1 (0.12)	0.139 (0.019 to 0.985)	<0.05	0.165 (0.023 to 1.175)	0.072
Intestinal perforation	8,743 (2.16)	9 (1.12)	0.513 (0.266 to 0.99)	<0.05	0.408 (0.182 to 0.912)	<0.05
Ileostomy	7,600 (1.87)	9 (1.12)	0.592 (0.307 to 1.142)	0.114	0.664 (0.315 to 1.4)	0.282
Colostomy	3,294 (0.81)	4 (0.49)	0.61 (0.228 to 1.63)	0.319	(0.28 to 2.005)	0.565
			Mean Difference (CI)	p-value	AOR (CI)	Adjusted p-value
Mean Length of Stay (SD)	7.82 days (10.691)	7.72 (8.573)	0.109±0.377 (-0.96 to 0.85)	<0.05	1.005 (0.997 to 1.013)	0.256
Mean Total Charges (SD)	$65,652.63 (113,213.548)	$47,557.67 (87,006.814)	18,094.962±4,043.126 (10,170.558 to 26,019.365)	<0.05	1 (1-1)	<0.05

Prevalence of IBD-associated arthropathy and erythema nodosum was zero in the HP group. The HP group had higher percentage of lower gastrointestinal bleed (10.19% versus 5.4%; p<0.05, OR 1.983, CI 1.577-2.492; adjusted p<0.05, AOR 1.972, ACI 1.525-2.551) with statistical significance before and after adjustment for confounders. There was no statistically significant difference in uveitis between the two groups (0.12% versus 0.04%; p=0.289, OR 2.77, CI 0.388-19.794; adjusted p=0.28, AOR 2.964, ACI 0.413-21.283). Non-HP patients had higher rates of intestinal perforation (2.16% versus 1.12%; p<0.05, OR 0.513, CI 0.266-0.99; adjusted p<0.05) with statistical significance before and after adjustment for confounding variables. These patients also had higher rates of primary sclerosing cholangitis (1.28% versus 0.37%; p<0.05, OR 0.288, CI 0.093-0.895; adjusted p=0.108, AOR 0.394, ACI 0.126-1.227), and intra-abdominal abscess (0.89% versus 0.12%; p<0.05, OR 0.139, CI 0.019-0.985; adjusted p=0.072, AOR 0.165, ACI 0.023-1.175) all with initial statistical significance prior to adjustment for confounders. Furthermore, there was no significant difference among the two groups when looking at bowel obstruction (2.17% versus 1.99%; p=0.728, OR 0.916, CI 0.558-1.503; adjusted p=0.786, AOR 1.073, ACI 0.643-1.793), ileostomy (1.87% versus 1.12%; p=0.114, OR 0.592, CI 0.307-1.142; adjusted p=0.282, AOR 0.664, ACI 0.315-1.4), and colostomy (0.81% versus 0.49%; p=0.319, OR 0.61, CI 0.228-1.63; adjusted p=0.565, AOR 0.749, ACI 0.28-2.005), both before and after adjustment for confounding variables (Table 2).

Non-HP patients had significantly higher total hospitalization charges ($65,652.63 versus $47,557.67; p<0.05, adjusted p<0.05). Length of stay was slightly higher among non-HP patients (non-HP: 7.82 days versus HP: 7.72 days; p<0.05), however statistical significance was lost after adjustment for confounders (adjusted p=0.256), shown in Table 2.

Most patients in both groups utilized Medicare (non-HP: 48.59% versus HP: 46.88%), followed by private insurance (non-HP: 37.62% versus HP: 31.3%), as seen in Table 3. In terms of median household income (Table 3), members of both groups (non-HP and HP) were predominantly in the highest quartile (52.21% versus 44.16%; p<0.05), followed by the third quartile (24.97% versus 28.57%). 

**Table 3:** Median household income & primary payer for ulcerative colitis patients with and without Helicobacter pylori infection n: sample size

		Ulcerative colitis without Helicobacter pylori		Ulcerative colitis with Helicobacter pylori		p-value
		Percentage	n	Percentage	n	
Primary payer	Medicare	48.59	196,625	46.88	376	<0.05
	Medicaid	7	28,349	11.35	91	
	Private insurance	37.62	152,246	31.3	251	
	Self-pay	3.55	14,350	5.61	45	
	No charge	0.4	1,602	0.5	4	
	Other	2.84	11,504	4.36	35	
Median household income	Lowest quartile	4.01	2,025	5.84	9	<0.05
	Second quartile	18.8	9,501	21.43	33	
	Third quartile	24.97	12,618	28.57	44	
	Highest quartile	52.21	26,382	44.16	68	

**Figure 1:**
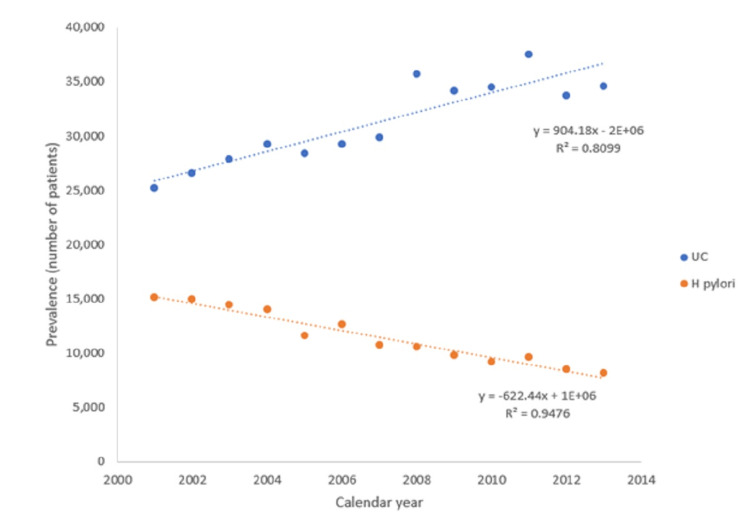
Yearly prevalence of ulcerative colitis (UC) and Helicobacter pylori (H. pylori) infection from 2001-2013

In terms of the individual yearly prevalence of ulcerative colitis and Helicobacter pylori (Table 4, Figure 1), rates of UC rose steadily from 2001 to 2013 by 37.38% from 25,169 to 34,578 (RR=0.8099; p<0.05). During the same time frame, rates of HP declined by 46.21% from 15,111 to 8,128 (RR=0.9476; p<0.05).

**Table 4:** Yearly prevalence of ulcerative colitis and Helicobacter pylori from 2001 to 2013 n: sample size

Calendar year	Ulcerative colitis			Helicobacter pylori		
	Percent of total cases from 2001-2013	n	p-value	Percent of total cases from 2001-2013	n	p-value
2001	6.2	25,169	<0.05	10.1	15,111	<0.05
2002	6.5	26,538		10	14,936	
2003	6.9	27,874		9.7	14,389	
2004	7.2	29,215		9.4	13,967	
2005	7	28,393		7.7	11,546	
2006	7.2	29,216		8.4	12,585	
2007	7.3	29,809		7.2	10,752	
2008	8.8	35,678		7.1	10,531	
2009	8.4	34,126		6.6	9,764	
2010	8.5	34,492		6.2	9,200	
2011	9.2	37,506		6.5	9,631	
2012	8.3	33,702		5.7	8,449	
2013	8.5	34,578		5.5	8,128	

## Discussion

In this study we explored the rate of complications in UC patients with HPI to better understand how the two conditions interact. We hypothesized that a physiologic basis for observed lower rates of HPI in UC patients would result in differences in disease severity, rather than just incidence. We found that over time, HP prevalence has decreased steadily while UC rates have progressively increased. We also found that patients with UC and HP had a lower mortality, hospitalization costs, and higher rates of intestinal perforation rates and lower gastrointestinal bleeding. Additionally, patients with UC and HPI had lower rates of intrabdominal abscess formation, although this difference was not present when accounting for potential confounders. Despite these differences, there was no difference in the rates of ileostomies and colostomies among patients with and without HP.

The inverse correlation between HP and UC is consistent with previous literature [11-13]. Furthermore, our results suggest that HP infections may have a protective effect for patients with UC, as demonstrated by the lower mortality rate, hospitalization cost, intestinal perforation, and higher rates of gastrointestinal bleeding. While some hypothesize that these differences in outcomes are secondary to differential geographic distribution of the two conditions, recent increases in IBD prevalence in Asia, where HP infection rates are high, suggest that varying geographic distributions may not play as much of a role as initially thought in terms of in-hospital outcomes [13-15]. Additionally, given the rise in inflammatory bowel disease globally, it is becoming more evident that environmental influences of the host microbiome play a larger part in IBD risk and phenotype than previously thought. As demonstrated in recent studies among patients with Crohn’s disease [15], and other autoimmune conditions [9,16,17], there may be an immunomodulatory effect that HP exhibits, reducing the overall severity of autoimmune disease such as UC, as seen in this study. For example, levels of antibodies against HP have been shown to be significantly lower in patients with autoimmune disease [9,16,17]. This relationship may be due to the immunomodulatory mechanisms used by HP to enable long-term infection, which would subsequently inhibit the immune response against autoantigens in addition to HP. Furthermore, HP has been shown to downregulate production of Th1 and Th17 cytokines and upregulate production of regulatory B-cells in mice [18,19]. These immune components are responsible for production of inhibitory cytokines such as interleukin 10 (IL-10) and transforming growth factor-β (TGF-β), which may lower the risk of developing clinically significant autoimmune disease [18,19].

Another hypothesis on the relationship between HPI and IBD suggests that the use of antibiotics to treat H. pylori may alter the gut microbiome, reducing diversity and impacting the severity of symptoms [20,21]. Studies have shown that germ-free mice, with their lack of microbiome diversity, often exhibit overexaggerated immune responses to bacterial antigens when compared to wild-type mice [22]. Furthermore, studies in humans have demonstrated that specific changes in microbiome diversity are linked with both UC and Crohn’s disease [23,24]. While there appears to be a prominent role for the microbiome in the development of IBD, the interaction is not fully elucidated, and further studies linked genetic and environmental factors are needed to clarify this relationship. However, given that H. pylori is treated with a relatively long course of multiple antibiotics, it is hypothesized that this may be altering the gut microbiome in a way that reduces biodiversity and thus increases the severity of IBD. This is supported Lin et al.’s study, which demonstrated an increased risk of developing autoimmune diseases, including IBD, following HP eradication [25].

There are some important limitations to our study. Firstly, NIS data relies on accurate coding of diagnoses. Additionally, the data does not provide a temporal relationship between the two conditions, making it difficult to draw conclusions on which condition is influencing the other. For example, separating patients who were treated for H. pylori vs. those who were not treated would provide useful information. Finally, we only analyzed hospitalizations in the United States, but as rates of ulcerative colitis increase in countries with a high prevalence of H. pylori, it will be important to analyze trends from these areas as well. 

## Conclusions

We explored complication rates of UC in patients with HPI to better clarify the interaction between the two conditions. Patients with UC and HPI had a lower mortality rate, hospitalization costs, and higher rates of intestinal perforation and lower GI bleeding. However, despite these differences, there was no difference in the rates of ileostomies and colostomies performed. Over time, HPI has decreased steadily while UC rates have increased steadily. Further studies are needed to better understand the role H. pylori plays in reducing the severity of UC.
